# Manipulating surface-plasmon-polariton launching with quasi-cylindrical waves

**DOI:** 10.1038/srep11331

**Published:** 2015-06-10

**Authors:** Chengwei Sun, Jianjun Chen, Wenjie Yao, Hongyun Li, Qihuang Gong

**Affiliations:** 1 State Key Laboratory for Mesoscopic Physics and Department of Physics, Peking University, Beijing 100871, China; 2 Collaborative Innovation Center of Quantum Matter, Beijing, China

## Abstract

Launching the free-space light to the surface plasmon polaritons (SPPs) in a broad bandwidth is of importance for the future plasmonic circuits. Based on the interference of the pure SPP component, the bandwidths of the unidirectional SPP launching is difficult to be further broadened. By greatly manipulating the SPP intensities with the quasi-cylindrical waves (Quasi-CWs), an ultra-broadband unidirectional SPP launcher is experimentally realized in a submicron asymmetric slit. In the nano-groove of the asymmetric slit, the excited Quasi-CWs are not totally damped, and they can be scattered into the SPPs along the metal surface. This brings additional interference and thus greatly manipulates the SPP launching. Consequently, a broadband unidirectional SPP launcher is realized in the asymmetric slit. More importantly, it is found that this principle can be extended to the three-dimensional subwavelength plasmonic waveguide, in which the excited Quasi-CWs in the aperture could be effectively converted to the tightly guided SPP mode along the subwavelength plasmonic waveguide. In the large wavelength range from about 600 nm to 1300 nm, the SPP mode mainly propagates to one direction along the plasmonic waveguide, revealing an ultra-broad (about 700 nm) operation bandwidth of the unidirectional SPP launching.

Surface plasmon polaritons (SPPs) are electromagnetic waves propagating along the metal and dielectric interface^1^,^2^,^3^. Because of their tight spatial confinements and strong local field enhancements, they are considered as one of the most competitive candidates of the next-generation information carriers. Over the past decade, enormous attention has been attracted, and varieties of nano-metallic structures have been proposed to guide and manipulate the SPPs^4^,^5^,^6^,^7^,^8^,^9^,^10^,^11^,^12^,^13^. In these processes, coupling the free space light to the SPPs on the metal surface as well as delivering the SPPs to the desired regions is of great importance for the development of plasmonic devices and systems because of the wave-vector mismatching. Therefore, many unidirectional SPP launchers were proposed and demonstrated in the experiments recently, such as the nanoslit with periodic grooves on one side^14^, the periodic ridges^15^, the aperiodic grooves^16^,^17^, the single asymmetric slit^18^, and two different interfering SPP sources^19^,^20^,^21^. However, because of the Bragg^14^,^15^,^16^,^17^ and interference^18^,^19^,^20^,^21^ conditions, these unidirectional SPP launchers are sensitive to the incident wavelengths, leading to a narrow-band operation. This greatly limits their applications in the future integrated circuits, which require broad operation bandwidths. In order to address this problem, broadband unidirectional SPP launchers^22^,^23^,^24^,^25^,^26^,^27^,^28^ were proposed and demonstrated, such as the dielectric-film-coated slit with chirped plasmonic crystal of grooves on one side^22^, chirped plasmonic crystal of slits^23^, two nano-grooves^24^ and nano-slits^25^ of different sizes, dielectric-film-coated asymmetric slit^26^, asymmetric optical slot nano-antennas^27^, and phased nanoslit pair^28^. These structures can realize unidirectional SPP launching with broad bandwidths of about 200 or 300 nm^22^,^23^,^24^,^25^,^26^,^27^,^28^.

In all of these metallic structures^14^,^15^,^16^,^17^,^18^,^19^,^20^,^21^,^22^,^23^,^24^,^25^,^26^,^27^,^28^, only the pure SPP component was mainly employed. This limits their further increasing of the operation bandwidths. It is well known that the quasi-cylindrical waves (Quasi-CWs)^29^,^30^,^31^,^32^ are also the main contribution to the total field near the metallic apertures (such as slits and holes) and play an important role in nanoscale structures and devices^33^,^34^,^35^,^36^,^37^. In most cases of the SPP launchers, the Quasi-CWs exhibit nearly the same properties (wave vector and initial phase) as the SPPs in the near field^31^ and they are completely attenuated at the detection ports in the experiments, and thus only the pure SPP component is considered^14^,^15^,^16^,^17^,^18^,^19^,^20^,^21^,^22^,^23^,^24^,^25^,^26^,^27^,^28^. In the letter, we propose to greatly manipulate the SPP launching with the Quasi-CWs in an asymmetric metallic slit. In the asymmetric single nanoslit, both of the SPPs and the Quasi-CWs can be excited in the nano-groove when a p-polarized beam illuminates it from the back side. Since the groove width is very small (<λ), the Quasi-CWs in the nano-groove are not totally attenuated^29^,^31^, and thus they can be scattered into the SPPs on the front metal surface by the metal wall of the groove (CW-to-SPP conversions)^31^,^32^. By adjusting the slit width, the SPPs scattered from the Quasi-CWs will interfere destructively with that scattered from the SPPs in the nano-groove. This greatly manipulates the SPP intensity along the front metal surface. Consequently, the propagation direction of the excited SPPs in the asymmetric nanoslit is altered, and broadband unidirectional SPP launching is realized theoretically and experimentally. This principle can be extended to 3D models, in which the Quasi-CWs near the aperture can be effective converted to the tightly guided SPP mode along the subwavelength plasmonic waveguide.

## Results

### Theoretical analysis

**Figure 1:**
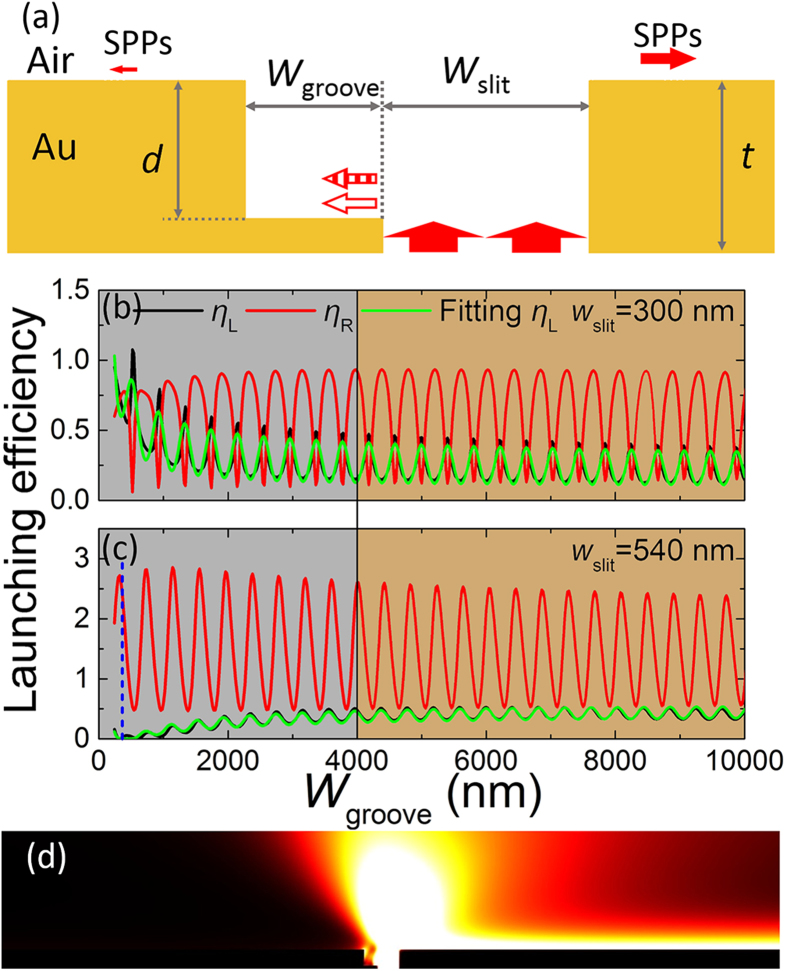
Schematic structure and simulation results of the asymmetric slit on the metal surface. (**a**) Structure of the asymmetric slit and the geometrical parameters. SPP launching efficiencies to the left (*η*_L_, black) and the right (*η*_R_, red) with changing the groove width for (**b**) *w*_slit_ = 300 nm and (**c**) *w*_slit_ = 540 nm when the groove depth is fixed to be *d* = 400 nm. The green lines are the fitting results using Eq. (1). (**d**) Power flow distribution of asymmetric structure for *w*_slit_ = 540 nm and *w*_groove_ = 360 nm.

The investigated asymmetric single nano-slit comprises a slit and an adjacent nano-groove on the gold film (the dimension in the *z* axis being infinite, 2D model), which is illuminated with a p-polarized beam from the back side, as schematically shown in Fig. 1. The thickness of the gold film is *t*. The slit width, groove width, and groove depth of the asymmetric slit are *w*_slit_, *w*_groove_, and *d*, respectively. The excited SPPs in the nano-groove can be reflected back and forth off the metal walls, and each round trip can contribute to the SPPs along the metal surface. Because of the large loss (Ohmic loss and scattering loss), completely destructive interference of the SPPs scattered from the different round trips in the nano-groove cannot be achieved^18^. Here, by utilizing the Quasi-CWs, which are also excited in the nano-groove by the nano-slit, the SPP launching is greatly manipulated, and both of the completely constructive and completely destructive interference can be obtained in the asymmetric slit.

To depict these two opposite cases in the asymmetric slit, the launching efficiencies of the SPPs in the opposite directions (*η*_L_ and *η*_R_) are simulated at λ = 830 nm, and the results are displayed in Fig. 1(b,c). Herein, the subscripts of L and R denote the left- and right-propagating directions of the SPPs. The calculated results are displayed in Fig. 1(b,c). In our simulation, the thickness of the gold film is *t* = 500 nm, and the groove depth is set to be *d* = 400 nm. The curves in Fig. 1(b,c) can be divided into two parts, as shown by the grey and brown areas. When the groove width is greater than about 4 μm, it is observed that both *η*_L_ and *η*_R_ present stationary periodic oscillation behaviors [brown areas in Fig. 1(b,c)]. The period is about 407 nm, which equals the half of the SPP wavelength (*λ*_SPPs_/2 = 830/1.019/2 = 407 nm). This agrees well with the analysis of the back and forth reflections of the SPPs in the groove^18^. When the groove width is smaller than about 4 μm [grey areas in Fig. 1(b,c)], the increasing [black line in Fig. 1(b)] and decreasing [black line in Fig. 1(c)] of the oscillation curves of *η*_L_ are observed for different slit widths. This indicates that the stationary oscillation behaviors are accompanied by additional interference. The constructive additional interference can increase the SPP launching efficiency^18^,^32^, as depicted by the black line in Fig. 1(b). Conversely, the destructive additional interference can greatly decrease the SPP launching efficiency in the left direction, as shown by the black line in Fig. 1(c). In this case, the SPP launching efficiency in the right direction nearly reaches the maximum value, as pointed by the blue dashed line in Fig. 1(c). The corresponding field distribution is displayed in Fig. 1(d), revealing that the excited SPPs by the asymmetric slit mainly propagate to the right direction. Here, the lateral dimension of the asymmetric slit is only about 900 nm. Therefore, the unidirectional SPPs can be launched in the submicron asymmetric slit.

**Figure 2:**
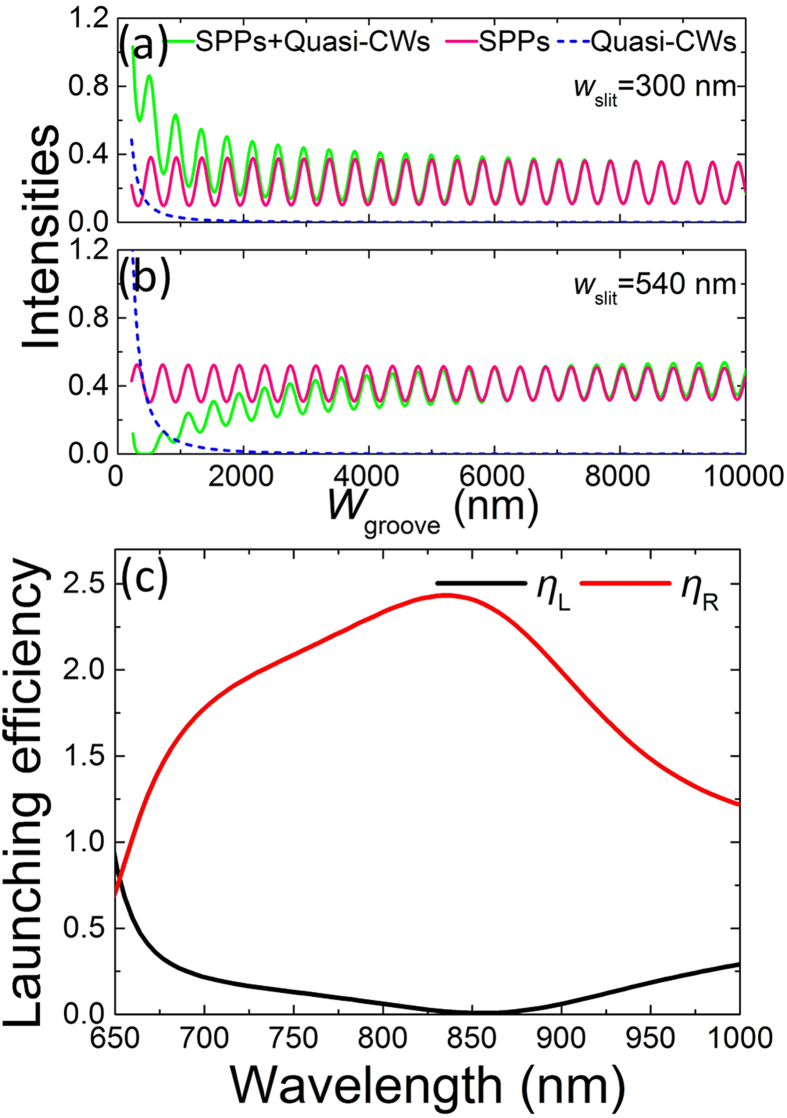
Contributions of the launching SPPs and the broadband operation property. Intensities of the left-propagating SPPs scattered from the SPPs (pink) and Quasi-CWs (blue) in the groove for (**a**) *w*_slit_ = 300 nm and (**b**) *w*_slit_ = 540 nm. The green lines are the fitting results using Eq. (1). (**c**) Simulation results of the launching spectra in the submicron asymmetric slit structure.

To understand the underlying physics of the additional interference for small groove widths, we study the grey areas in Fig. 1(b,c), where the interfering states of the left-propagating SPPs have a relationship with both of the slit width and groove width. As mentioned above, when the groove width is small, the Quasi-CWs in the groove are not totally attenuated^29^,^31^, and they can be scattered into the left-propagating SPPs^32^ by the left metal wall. Hence, the left-propagating SPPs along the front metal surface originate from the interference of two contributions. One is scattered from the SPPs of different round trips in the groove, and the other is scattered from the Quasi-CWs in the groove. As a result, the amplitude of the left-propagating SPPs excited by the asymmetric slit is determined by


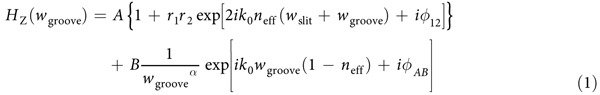


where, *r*_1_*r*_2_ is the product of the amplitudes of the reflectivity off the left and right groove walls; *n*_eff_ = 1.0193 + 0.0012*i* is the effective refractive index of the SPPs on the front metal surface; *k*_0_ = 2π/λ is the wave vector in vacuum; *A* and *B* are the amplitudes of the SPPs on the front metal surface scattered from the SPPs and Quasi-CWs in the groove when *w*_groove_ approaches zeros, and the values of *A* and *B* are determined by *w*_slit_ and *d*; *φ*_AB_ is the initial phase difference between the SPPs that are scattered from the SPPs and Quasi-CWs in the groove, and it can be varied by adjusting the structural parameters^32^; *α* is the average attenuation constant of the Quasi-CWs^29^,^31^; and *φ*_12_ is the phase brought by the reflections off the groove walls. The power flow of the SPPs is proportional to the |*H*_z_|^2^. To eliminate the influence of the detection port position on the simulation and experimental results, *H*_z_ in Eq. (1) has been normalized by exp[−*L*/(2*L*_SPP_)], where *L* and *L*_SPP_ are the slit-detection-port spacing and the SPP propagation length, respectively. Here, it should be pointed out that Eq. (1) is based on two main simplifications. The first one is that only one round-trip reflection of the SPPs in the groove is considered due to the large loss (Ohmic loss and scattering loss in the groove)^18^, while no round-trip reflection of the Quasi-CWs is included because the Quasi-CWs decay much faster than the SPPs^29^,^31^ in the groove. The second one is that the average attenuation constant of the Quasi-CWs is chosen for the sake of convenience^31^. To test the validity of Eq. (1), the fitting results for the left-propagating SPPs using Eq. (1) are displayed by the green lines in Fig. 1(b,c). A good agreement between the simulation results and the fitting results is observed, strongly confirming our analysis. In the fitting, we obtain *α* ≈ 0.903, which is very near the fitting value of 0.872 at λ = 800 nm in Ref. 31. For *w*_slit_ = 300 nm, it is got that *r*_1_*r*_2_ ≈ 0.334, *φ*_12_ ≈ −0.00958π, and *φ*_AB_ ≈ −0.0989π. Here, *φ*_AB_ ≈ −0.0989π indicates that the first and second parts in Eq. (1) have the same signs. Thus, nearly completely constructive interference occurs, resulting in the increasing of the launching efficiency, as shown by the black line in the grey area of Fig. 1(b). For *w*_slit_ = 540 nm, it is got that *r*_1_*r*_2_ ≈ 0.137, *φ*_12_ ≈ −0.120π, and *φ*_AB_ ≈ 0.803π. Here, *φ*_AB_ ≈ 0.803π reveals that the first and second parts in Eq. (1) have the opposite signs. Thus, nearly completely destructive interference happens, leading to the great decreasing of the launching efficiency, as shown by the black line in the grey area of Fig. 1(c). To clearly demonstrate the contributions from the SPPs and Quasi-CWs in the groove, the intensities of the two parts in Eq. (1) are depicted in Fig. 2(a,b). It is observed that the first part (pure SPP component) exhibits stationary oscillation behaviors (pink lines), and the completely destructive interference of the SPPs from the different round trips in the nano-groove cannot be obtained (pink lines) because of the large loss (Ohmic loss and scattering loss)^18^,^38^. For the second part, which is scattered from the Quasi-CWs in the groove (without considering the round-trip reflection of the Quasi-CWs), it exhibits attenuation behaviors [blue dashed lines in Fig. 2(a,b)]^29^,^31^. When the two parts in Eq. (1) have the same signs, the constructive interference occurs, leading to the increasing of the launching efficiency in the near field, as shown by the green line in Fig. 2(a). On the contrary, when the two parts in Eq. (1) have opposite signs, the destructive interference would emerge, resulting in the decreasing of the launching efficiency in the near field, as shown by the green line in Fig. 2(b). This matches well with the above analysis. By utilizing the Quasi-CWs, the left-propagating SPPs extinct, and the excited SPPs mainly propagate to the right direction, as shown in Fig. 1(d). Thus, the unidirectional SPP launching is realized in the asymmetric slit. For the right direction, the reflected Quasi-CWs can also be scattered into the right-propagating SPPs by the right metal wall^32^. But, this process has little influence on the launching efficiency of the right-propagating SPPs [the red lines in Fig. 1(b,c)] because of the small reflectivity of the Quasi-CWs off the left groove wall and large decaying rate of the Quasi-CWs^31^.

The extinction ratio of the unidirectional SPPs is mainly dominated by the SPP launching efficiency in the extinction direction. Based on Eq. (1), the period of the additional interference referring to the incident wavelengths is about ∆λ = λ^2^/[(*n*_eff_ − 1)*w*_groove_] ≈ 1 × 10^5^ nm, which is much greater than ∆λ = λ^2^/(2*n*_eff_*w*_groove_) ≈ 9 × 10^2^ nm based on the pure SPP model. Hence, by utilizing the Quasi-CWs, a broadband unidirectional SPP launcher can be achieved in the submicron asymmetric slit. To explore the bandwidth operation property of the unidirectional SPP launcher based on the additional interference, the launching spectra are calculated for *w*_slit_ = 540 nm and *w*_groove_ = 360 nm, and the results are shown in Fig. 2(c). From Fig. 2(c), it is observed that the *η*_R_ is much greater than *η*_L_ in the walvelength ranges from 680 nm to 980 nm, which leads to a broadband unidirectional SPP launcher.

### Experimental demonstration of the ultra-broadband SPP launcher on a planar metal film

**Figure 3:**
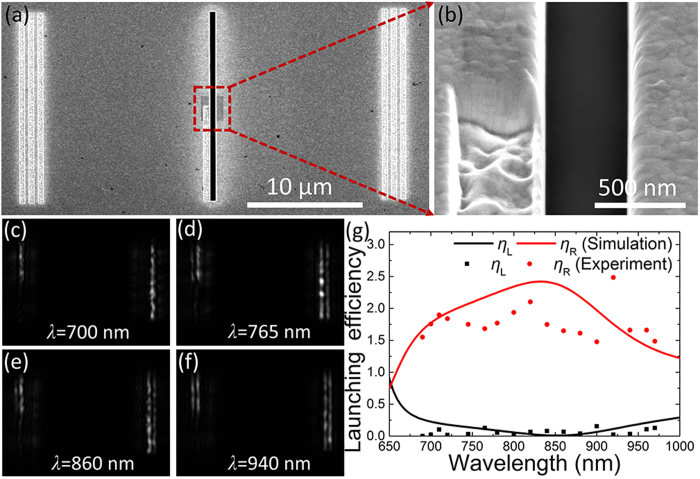
Experimental demonstration of the broadband unidirectional SPP launching in the asymmetric slit. (**a**) SEM image of the experimental sample on the Au film. (**b**) Detail of the asymmetric slit. Scattered field distributions for different incident wavelengths of (**c**) *λ* = 700 nm, (**d**) *λ* = 765 nm, (**e**) *λ* = 860 nm, and (**f**) *λ* = 940 nm. (**g**) Both the left- (black) and right-propagating (red) SPP launching efficiencies versus the wavelengths obtained in the simulation (solid line) and experiment (symbols).

To test our proposal experimentally, the asymmetric slit is fabricated on a planar metal film, and the scanning electron microscope (SEM) image of the experimental sample is shown in Fig. 3(a,b). The measured geometrical parameters of the fabricated asymmetric slit structure are about: *w*_slit_ = 540 nm, *w*_groove_ = 360 nm, and *d* = 400 nm. So the total lateral dimension is only about 900 nm. An in-chip reference slit is also fabricated for comparison. In the experiment, it is observed that the scattering light from the upper parts of the decoupling gratings is nearly the same because of the structural symmetry of the in-chip reference slit. However, for the lower parts of the decoupling gratings, the phenomena are quite different. We observed that the left grating is nearly dark while the right one keeps bright in a broad bandwidth, and the extinction ratio is greater than 11 dB, as show in Fig. 3(c–f). This indicates the excited SPPs mainly propagate to the right direction. Moreover, the lower right grating is always brighter than the upper gratings, revealing that the launching efficiencies increase greatly. Figure 3(g) depicts the measured launching efficiency, *η*, at different wavelengths (dots). Here, *η* is obtained from the quotient between the light intensities scattered from the lower and the upper parts of each decoupling gratings (evaluated by integration over a spatial scale on the grating)^18^. By doing this, the laser fluctuation and the CCD sensitivity varying with the wavelengths can be eliminated. From Fig. 3(g), it is observed that *η*_R_ (red dots) is always greater than 1.5 while *η*_L_ (black dots) is nearly vanished within the measured wavelength range from 675 nm to 970 nm, which matches the simulation results (solid lines) quite well. Therefore, a broadband unidirectional SPP launcher with increasing launching efficiencies and high extinction ratios is realized in the submicron asymmetric slit structure.

### Ultra-broadband SPP launcher in a subwavelength plasmonic waveguide

**Figure 4:**
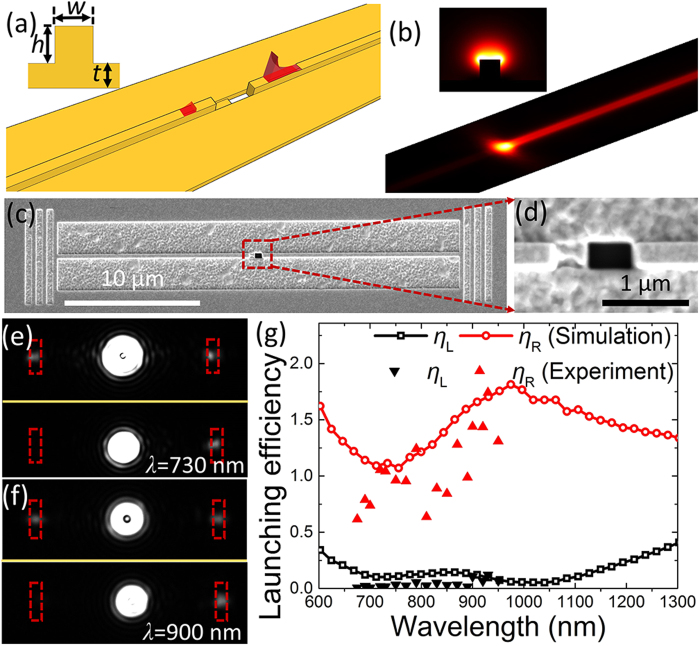
Ultra-broadband SPP launcher beyond diffraction limit in a subwavelength plasmonic waveguide. (**a**) Schematic structure of the asymmetric aperture fabricated in a subwavelength plasmonic waveguide. Inset shows the cross section of the plasmonic waveguide. (**b**) Power flow distribution of the excited SPPs on the cross section 100 nm above the plasmonic waveguide. Inset shows the field distribution of the SPP mode supported by the subwavelength plasmonic waveguide. (**c**) SEM image of the experimental sample. (**d**) Detail of the asymmetric aperture fabricated in the plasmonic waveguide. CCD images of the reference (top) and experimental sample (bottom) at (**e**) λ = 730 nm and (**f**) λ = 900 nm. (**g**) Experimental (solid symbols) and simulation (lines with hollow symbols) results of both the left- (black) and right-propagating (red) SPP launching efficiencies versus the wavelengths.

The manipulation of SPPs by utilizing the Quasi-CWs on the planar metal surface (2D) can be extend to the subwavelength plasmonic waveguides (3D), which can significantly shrink the device below the diffraction limit as well as avoid the large crosstalk between different plasmonic devices in the plasmonic circuits. Here, the submicron asymmetric slit is fabricated on a subwavelength plasmonic waveguide instead of the metal surface, as schematically shown in Fig. 4 (a). The inset shows the cross section of the plasmonic waveguide, which comprises a 300 × 300 nm^2^ gold ridge on a 200 nm-thick gold film. Fig. 4(b) displays the simulation result of the field distribution in the proposed structure, where the excited SPPs mainly propagate to the right direction along the subwavelength plasmonic waveguide, just like the 2D case. The inset in Fig. 4(b) shows the field distribution of the SPP mode supported by the subwavelength plasmonic waveguide, revealing that the SPPs are well confined by the plasmonic waveguide. Experimentally, such a structure is fabricated by FIB on the same gold film, and the SEM image of the sample is shown in Fig. 4(c,d). The measured structural parameters of the asymmetric slit are about: *w*_slit_ = 500 nm, *w*_groove_ = 400 nm, and, *d* = 400 nm. The cross section of the gold ridge is about 300 × 300 nm^2^. So it only occupies a footprint of about 0.27 μm^2^ on the subwavelength plasmonic waveguide. We also fabricated an in-chip reference, in which only a 500 nm-wide rectangular aperture is fabricated on the subwavelength plasmonic waveguide.

In the measurement, it is observed that the broadband unidirectional SPP launching is still maintained, very like the 2D case on the planar metal surface. The CCD pictures at two typical incident wavelengths are shown in Fig. 4(e) (λ = 730 nm) and Fig. 4(f) (λ = 900 nm), where the upper and lower parts are the reference sample and experimental sample, respectively. From these two figures, we can observe that the decoupling gratings of the reference sample (top) have the same brightness on both sides because of the structural symmetry. However, for the experimental sample (bottom), it is clearly seen that the right decoupling grating is bright while the left one is very dark. The measured extinction ratios are about 17 dB and 11 dB for *λ* = 730 nm and *λ* = 900 nm, respectively. Hence, the unidirectional launching of the SPPs is achieved in the asymmetric aperture beyond the diffraction limit. More importantly, only the middle parts of the decoupling gratings are lighted up [Fig. 4(e,f)], which reveals the excited SPPs are well confined and guided along the subwavelength plasmonic waveguide. The launching efficiencies versus wavelengths obtained in the experiment and simulation are shown in Fig. 4(g). Due to the limitation of the tunable wavelength range of the laser and the response of the CCD, we only measured the launching efficiency in the range from 675 nm to 950 nm [solid symbols in Fig. 4(g)]. A well agreement between the experiment and simulation is observed. Moreover, it is noted that *η*_R_ is much greater than *η*_L_ in the wavelength ranges from about 600 nm to 1300 nm [see the simulation results in Fig. 4(g)]. This is an ultra-broad (about 700 nm) operation bandwidth for the unidirectional SPP launching. Therefore, it can be concluded that the CW-to-SPP conversion also exists in the subwavelength plasmonic waveguide. This can be used to greatly manipulate the SPP intensity in the subwavelength plasmonic waveguides. This ultra-broadband (about 700 nm) unidirectional SPP launcher beyond the diffraction limit has important applications in the high-density plasmonic circuits.

## Discussion

Although the SPPs dominate the Quasi-CWs in the visible regime (short wavelength)^29^, the conversion efficiency of CW-to-SPP becomes large at short wavelengths^32^, and the transmittance of the original SPPs in the 400-nm-deep groove to the SPPs along the front metal surface (SPP-to-SPP) becomes very small (10% at λ = 650 nm for the 400-nm-deep groove on the metal surface). Taking both of these factors into account, the dominant original SPPs with low transmittance can interfere destructively with the SPPs converted from the small fraction of Quasi-CW with high conversion efficiency. Therefore, directional launching can be obtained in the visible regime. At long wavelengths (near-infrared regime), the fraction of Quasi-CW increases, but the conversion efficiency of CW-to-SPP decreases^32^ and the transmittance of the original SPPs in the 400-nm-deep groove increases (50% at λ = 1100 nm for the 400-nm-deep groove on the metal surface). As a result, the destructive interference between the SPPs converted from the Quasi-CW and the original SPPs can also be realized at long wavelengths. Therefore, directional launching can be obtained in a very broad bandwidth.

By utilizing the Quasi-CWs in the near field, a broadband unidirectional SPP launcher was theoretically and experimentally realized in the asymmetric slit structure. For a short groove width, the Quasi-CWs in the nanogroove were not totally attenuated, and they could be scattered into the SPPs along the metal surface by the metal wall. This greatly manipulated the left-propagating SPP intensities. By adjusting the slit width, the completely destructive interference between the left-propagating SPPs coming from the SPPs and Quasi-CWs in the groove happened. This resulted in that the excited SPPs mainly propagated to the right direction. Hence, a broadband unidirectional SPP launcher was realized theoretically and experimentally. This 2D principle can be extended to the 3D geometries, in which the excited Quasi-CWs near the aperture could be converted to the tightly guided SPP mode along the subwavelength plasmonic waveguide. This resulted in that the intensity of the right-propagating SPPs is much greater than that of the left-propagating SPPs in the wavelength ranges from about 600 nm to 1300 nm, revealing an ultra-broad operation bandwidth (about 700 nm) of the unidirectional SPP launcher comparing with the previous results (from about 200 to 300 nm)^22^,^23^,^24^,^25^,^26^,^27^,^28^. The great control of the SPP intensity with the Quasi-CWs in the near field can provide a new possibility to manipulating the light in the subwavelength scales.

## Methods

### Simulation

The launching efficiencies of the SPPs are calculated with the commercial software package of COMSOL Multiphysics. To calculate the SPP power flow, we set two 200-nm-high detection ports, which are 15 μm away from the center of the slit at the front metal surface. The launching efficiencies are obtained from the quotient between the power flows integrated at the detection ports in the slit structure with and without the nanogroove^18^,^25^,^26^,^38^. In the simulation, the permittivity of the air and gold are *ε*_Air_ = 1.0 and *ε*_Au_ = −26.61 + 1.665i^39^ at λ = 830 nm, respectively. The permittivity of gold for different wavelengths is taken from literature^39^ and expanded with the interpolation method.

### Fabrication

The asymmetric slit is fabricated using the focused ion beams (FIB) on a 450-nm-thick gold film, which is evaporated on a 30-nm-thick Ti-adhesion layer. First, a 20-μm-long slit is fabricated on the gold film. Then, a 10-μm-long groove is fabricated next to the lower half of the slit, constructing the asymmetric slit. The upper part is a symmetric slit, acting as an in-chip reference. Finally, two gratings (period of 800 nm and separation of 30 μm) are fabricated on both sides of the slit. For the asymmetric slit in the subwavelength plasmonic waveguide, two 30-μm-long and 2-μm-wide grooves are fabricated on the gold film first. Then, a rectangular aperture is fabricated on the center of the plasmonic waveguide. Last, a rectangular groove is fabricated next to the rectangular aperture, constructing the asymmetric aperture structure. Two 6 μm-long gratings (period of 800 nm and separation of 31.2 μm) are also fabricated on both sides to scatter the SPPs to the free space for the far-field detection.

### Measurement

A p-polarized laser beam (Ti: sapphire laser) with a radius of about 100 μm illuminates the sample normally from the back side. The SPPs excited by the asymmetric slit propagate along the front metal surface before being scattered to the free space by the decoupling gratings. The scattering light is collected by a long working distance objective lens (Mitutoyo, 50×, NA = 0.55) and then imaged onto a charge coupled device (CCD). The direct transmission light from the slit is blocked to prevent the CCD from saturation, so only the light scattered from the two gratings are imagined onto the CCD.

## Additional Information

**How to cite this article**: Sun, C. *et al.* Manipulating surface-plasmon-polariton launching with quasi-cylindrical waves. *Sci. Rep.* **5**, 11331; doi: 10.1038/srep11331 (2015).
